# Mild behavioral impairment is longitudinally associated with frailty in very old adults with MCI: insights from COGFRAIL.

**DOI:** 10.1007/s40520-025-03309-9

**Published:** 2026-02-03

**Authors:** Astrid Sawicki, Emmanuel Gonzalez-Bautista, Alberta Peluso, Gabor Abellan van Kan, Sandrine Sourdet, Maria Soto

**Affiliations:** 1 Gérontopôle de Toulouse. CHU Toulouse, IHU HealthAge, 20 Rue du Pont Saint-Pierre, 31300 Toulouse, France; 2 Clinical and Research Alzheimer’s Disease Center (CMRR), CHU Toulouse, IHU HealthAge, Toulouse, France; 3 Maintain Aging Research team, CERPOP UMR 1295 Inserm, Université de Toulouse, Toulouse, France

**Keywords:** Frailty, Mild behavioral impairment, Neuropsychiatric symptoms, Gait speed

## Abstract

**Background:**

The link between two often overlapping entities: mild behavioral impairment (MBI) and frailty has not been fully understood.

**Aim:**

We aimed to investigate the longitudinal association between MBI and frailty.

**Methods:**

Secondary analysis of COGFRAIL (clinicaltrials.gov NCT03129269). Real-life participants from the Toulouse Frailty Clinic with mild cognitive impairment were followed up for two years. MBI was measured using the NPI-Q. Frailty was defined by gait speed (GS) and Fried’s phenotype. We used mixed-effects models to analyse the longitudinal change in gait speed according to the presence and severity of MBI domains, and we examined the risk of incident frailty according to MBI status, using Cox models.

**Results:**

n **=** 234, mean age 83 SD ± 5.3, 58% (*n* = 135) were pre-frail at baseline. 12% were MBI-free. Participants with decreased motivation at baseline had a steeper decline in GS (*p* = 0.037), with higher severity directly associated with steeper decline (2-year mean decline of -0.047 m/s, 95% CI -0.092; -0.002). Similar results were found for the abnormal perception domain (2-year mean decline of -0.093 m/s, 95% CI: -0.155 to -0.032). Each higher severity points for decreased motivation at baseline resulted in a 33% higher risk of incident frailty than their counterparts over a mean follow-up of 284 days (SD 346), if measured at baseline HR = 1.33, (95% CI 1.07; 1.66), and as a time-varying variable HR = 1.39 (IC 95% 1.12; 1.72).

**Conclusions:**

The MBI domains of decreased motivation and abnormal perception were longitudinally associated with a steeper decline in gait speed, and decreased motivation predicts incident frailty.

**Supplementary Information:**

The online version contains supplementary material available at 10.1007/s40520-025-03309-9.

## Introduction

Mobility limitations and behavioral changes often overlap in the clinical presentation of older adults [1]; however, it is unclear whether these factors are connected. In this context, frailty—a clinical syndrome characterized by low physiological reserves in older adults—has significant implications for functional capacity, particularly in cognitive and physical domains, and is often associated with accelerated functional decline.

Frailty can be evaluated using different approaches, such as gait speed as an indicator of mobility reserves [2] and Fried’s phenotype [3]. Beyond physical aspects, psychological factors are linked to frailty, particularly when they co-occur with cognitive decline [4]. This depletion of psychological reserves is also seen in cases of mild behavioral impairment. (MBI) [5].

MBI is defined as persistent late-onset (age 50 and over) behavioral and psychological changes, different from primary psychiatric disorders, in dementia-free people, which have shown to be predictors of both cognitive and functional decline and a faster dementia conversion [6–9]. MBI is relevant as it can evoke an organic brain disease that might otherwise be misinterpreted as purely psychiatric [10].

MBI includes five different domains [6] : (1) decreased motivation, characterized by a loss of interest in previously enjoyable activities; (2) emotional dysregulation manifests as persistent sadness, depressive symptoms, excessive or inappropriate anxiety, and irritability, (3) impulse dyscontrol involves difficulties with regulating impulses, leading to aggression, verbal outbursts, or increased risk-taking tendencies, (4) social inappropriateness reflects tactlessness, diminished empathy, and (5) abnormal perceptions is marked by delusions, paranoid thoughts, or hallucinations.

The interplay between MBI and frailty has not been sufficiently explored. A few cross-sectional studies have been published, identifying higher frailty scores in individuals with MBI domains, including diminished motivation, affective dysregulation, and abnormal perception. However, questions such as the directionality, the MBI-domain-specific link to frailty, and the consistency of the association across different frailty metrics remain unanswered [11–13]. These gaps emphasize the need for research examining how MBI might predict changes in frailty over time. Indeed, our study adds value over existing cross-sectional studies by analyzing the longitudinal relationship between these two entities, and in particular how the MBI could predict frailty over time. This is an interesting factor in preventing functional decline. Furthermore, our study adds information on the very old, an often underrepresented age group in studies with a pre-dementia focus.

In this study, we aim to investigate the longitudinal association between MBI and frailty in a geriatric population without dementia in order to assess the significance of this relationship and better determine the impact of MBI on frailty. We hypothesize that MBI and its distinct domains may be longitudinally associated with the progression of frailty, as measured by gait speed and incidence of frailty based on Fried’s criteria.

## Methods

### Study design

We conducted a secondary analysis using longitudinal data from the Cognitive Function and Amyloid Marker in Frail Older Adults (COGFRAIL) study, a single-center, prospective observational study involving a clinical cohort of older adults. Between January 2017 and February 2020, volunteers were invited to participate in COGFRAIL during routine visits to the Frailty Clinic or Memory Clinic at Gérontopôle, Toulouse University Hospital, or through community care assessments. The participants were followed up every 6 months for 2 years after enrolment. The study aimed to investigate the prevalence of cerebral amyloid pathology and comprehensively track cognitive and physical changes over two years. The COGFRAIL study received approval from the institutional research committee (RC31/16/8753; clinicaltrials.gov NCT03129269). All participants provided written informed consent, adhering to the principles of the Declaration of Helsinki.

### Study population

The baseline cohort of COGFRAIL included 317 older adults (aged 70 years or above) with objective cognitive impairment and being prefrail or frail according to Fried’s phenotype. Eligibility criteria required participants to have a Clinical Dementia Rating (CDR) global score of 0.5 (mild cognitive impairment) or 1.0 (mild dementia) and a Mini-Mental State Examination (MMSE) score of > 20. Exclusion criteria included severe clinical or psychological conditions such as cancer or severe depression, dependency on more than two activities of daily living (bathing, dressing, toileting, transferring, continence, or feeding, with a Katz Index score ≥ 4), and legal restrictions such as being under judicial protection, guardianship, or supervision. Further details have been published elsewhere [14]. For this study, 31 participants from the baseline sample (*n* = 317) were excluded due to missing data for the MBI variables, and 52 participants with a baseline CDR ≥ 1 were also excluded, resulting in a final sample size of 234 participants.

### Frailty assessment

1) Gait speed, as a continuous variable to study its changes through time, was measured as the usual gait speed of participants across a distance of 4 m. We recorded the variable as the equivalent in meters per second (m/s) [15, 16].

2) Frailty based on Fried’s phenotype criteria [3] *Weakness*: sex- and body mass index (BMI)-adjusted grip strength measured with a Jamar hand dynamometer, considering the best result of two measurements. *Slowness*: predefined gait speed cut-off points based on height and walking pace over a 4-meter distance. *Exhaustion*: self-reported fatigue, assessed through two questions from the Center for Epidemiologic Studies Depression Scale (CES-D Scale): “During the last week, how often did you have experienced the following feeling: << everything I have done has required an effort > > and < < I cannot keep going like this>>” (options: rarely, sometimes, often, most of the time), considering the patient exhausted if the options “often” or “most of the time” were selected for at least one of the two questions. *Unintentional weight loss*: involuntary loss of more than 4.5 kg or more than 5% of body weight within the past year. *Low physical activity*: self-assessed using a scale developed initially by Saltin and Grimby [17] and later modified by Mattiasson-Nilo et al. [18]. This classification system includes both physical exercises and daily activities, consisting of six grades: (1) hardly any physical activity, (2) mostly sedentary, with occasional walks, light gardening, or household tasks like heating food, dusting, or tidying up, (3) Light physical activity for about 2–4 h per week, such as walking, fishing, dancing, regular gardening, or walks to and from stores, along with light domestic duties like cooking, cleaning, and making beds, (4) Moderate activity for 1–2 h per week, such as jogging, swimming, heavy gardening, home repairs, or more than 4 h of light activity per week, with full responsibility for all household chores, including vacuuming, washing floors, and window cleaning, (5) Moderate exercise for at least 3 h per week, such as tennis, swimming, or jogging, (6) Intense or very intense exercise several times a week, such as jogging or skiing. The “low physical activity” category was applied to those rated 1 or 2.

Frailty was assessed at baseline and 12- and 24-month follow-up. Participants without any criteria were considered robust (even if there were none at baseline, reversion was possible during follow-up); those with fewer than 3 criteria were considered pre-frail, and those with 3 or more criteria were considered frail. Among pre-frail participants, incident frailty was considered at the time of the first frailty record.

### Mild behavioral impairment (MBI) assessment

Following the ISTAART-AA research diagnostic criteria [6], we excluded participants with a CDR score of 1 or higher. We used a previously published algorithm [19, 20] to approach the MBI-C domains using the Neuropsychiatric Inventory Questionnaire (NPI-Q) [21]. The NPI-Q evaluates the status and severity of ten neuropsychiatric symptoms (NPS) (delusions, hallucinations, agitation/aggression, dysphoria/depression, anxiety, irritability, disinhibition, euphoria, apathy, aberrant motor behavior) and two neuro vegetative domains (sleep and night-time behavior, and appetite/eating), one item each, with a severity scoring from 1 (mild) to 3 (severe). We operationalized the five MBI domains as follows: decreased motivation (NPI-Q apathy/indifference); emotional/affective dysregulation (NPI-Q depression/dysphoria, anxiety, elation/euphoria); impulse dyscontrol (NPI-Q agitation/aggression, irritability liability, aberrant motor behavior); social inappropriateness (NPI-Q disinhibition); and abnormal perception or thought content (NPI-Q delusions, hallucinations). The NPI-Q was completed by a family member or close informant at baseline, 12, and 24 months.

*MBI domain status* was defined as present if at least one of its composing NPI items was positive.

*MBI domain severity* was defined as the arithmetic addition of the severity scores from the NPI-Q items (e.g., the severity of the MBI domain of abnormal perception may range from 0 to 6, given that it encompasses the two NPI-Q items of delusions and hallucinations).

### Covariates

Age (by birth date), self-reported sex, higher educational level, MMSE score [22], total body weight objectively measured, self-report of history of major depressive disorder, number of comorbidities from the following: hypertension, diabetes, heart failure, hypercholesterolemia, chronic kidney disease, chronic obstructive pulmonary disorder, arthritis, cancer; prescription of psychotropics (i.e., antidepressants, antipsychotics, mood-regulators, hypnotics).

### Statistical analyses

We used conventional descriptive statistics, including means and standard deviations (SD) for continuous variables, as well as numbers (n) and percentages (%) for categorical variables. The MBI domains were introduced in the models with their baseline values and in separate models as time-varying to maximize the period sensitive to MBI detection. The latter was done to address the fact that the use of the NPI-Q shortens the reference period to one month instead of the six-month reference period for the MBI-C.

We used linear mixed-effect models with random intercept (adjusting for baseline gait speed) at the individual level to analyze the longitudinal change in gait speed according to MBI status and severity by domain. Besides the primary exposure variable and the covariates, the models included the time variable and the interaction with time. The latter coefficient measures the change in gait speed (in m/s) per month of observation (Table 2). When the coefficients are significantly different from zero, the change in gait speed through time differs between participants with the presence versus absence of the MBI domain or across levels of MBI domain severity. We used the marginal means obtained from the adjusted models to plot the difference in gait speed decline across groups of interest. Additionally, we explored age as an effect modifier of the MBI-frailty association by including an MBI x time x age interaction in the mixed effect models.

To estimate the risk of incident frailty based on MBI status, we used Cox proportional hazards models for non-recurrent events, excluding frail participants at baseline. The visit date served as the time variable, so the time to event was measured in days. We verified the proportional hazards assumption using the Schoenfeld residuals test and found that the predictor-time interactions were not statistically significant at α = 0.05. Kaplan-Meier curves are provided for the model with significant results. All analyses were performed in Stata Statistical Software: Release 18. College Station, TX: StataCorp LLC.

## Results

Participants, *n* = 234 had a mean age of 83 (SD 5.3; range 70–98), and 63.2% were female. The mean MMSE score was 24.6 (SD 2.8); they averaged 2 comorbidities (SD 1.3), with a history of depression prevalent in 28.6% of participants and a 36.6% prevalence of psychotropic prescription (Table 1). At baseline, 58% (*n* = 135) were pre-frail, and 42.3% (*n* = 99) were frail. There were no participants with robust or CDR = 0 scores at baseline due to the inclusion criteria.

**Table 1:** Description of the population

Variable	70–79	80+	Total
N	71 (30.3%)	163 (69.7%)	234 (100.0%)
Age	76.4 (2.7)	85.7 (3.4)	82.9 (5.3)
Female	48 (67.6%)	100 (61.3%)	148 (63.2%)
Education			
Primary or less	28 (39.4%)	54 (33.1%)	82 (35.0%)
Mid school	31 (43.7%)	80 (49.1%)	111 (47.4%)
University or higher	12 (16.9%)	29 (17.8%)	41 (17.5%)
MMSE	24.8 (2.9)	24.8 (2.8)	24.8 (2.8)
CDR 0.5	71 (100%)	163 (100%)	234 (100%)
Weight	71.5 (14.6)	65.9 (12.7)	67.6 (13.5)
Comorbidities	1.9 (1.3)	2.2 (1.3)	2.1 (1.3)
Psychotropic treatment	31 (44.3%)	52 (33.1%)	83 (36.6%)
Gait speed	0.83 (0.23)	0.82 (0.20)	0.82 (0.21)
History of depression	31 (43.7%)	36 (22.1%)	67 (28.6%)
MBI domains presence^#^			
Decreased motivation	44 (62.0%)	76 (46.6%)	120 (51.3%)
Affective dysregulation	56 (78.9%)	118 (72.4%)	174 (74.4%)
Impulse dyscontrol	36 (50.7%)	86 (52.8%)	122 (52.1%)
Social Inappropriateness	14 (19.7%)	34 (20.9%)	48 (20.5%)
Abnormal perception	9 (12.7%)	29 (17.8%)	38 (16.2%)
MBI domain severity^#^			
Decreased motivation	1.0 (0.9)	0.8 (1.0)	0.9 (1.0)
Affective dysregulation	2.1 (1.7)	2.0 (1.8)	2.0 (1.8)
Impulse dyscontrol	1.4 (1.7)	1.4 (1.8)	1.4 (1.8)
Social Inappropriateness	0.3 (0.7)	0.3 (0.7)	0.3 (0.7)
Abnormal perception	0.1 (0.4)	0.3 (0.8)	0.2 (0.7)
Number of MBI domains impaired^#^			
0	4 (5.6%)	25 (15.3%)	29 (12.4%)
1	16 (22.5%)	30 (18.4%)	46 (19.7%)
2	21 (29.6%)	42 (25.8%)	63 (26.9%)
3	21 (29.6%)	41 (25.2%)	62 (26.5%)
4	7 (9.9%)	19 (11.7%)	26 (11.1%)
5	2 (2.8%)	6 (3.7%)	8 (3.4%)
Frailty phenotype			
Prefrail	39 (54.9%)	96 (58.9%)	135 (57.7%)
Frail	32 (45.1%)	67 (41.1%)	99 (42.3%)
Mean number of Fried’s frailty criteria	2.5 (1.2)	2.3 (1.0)	2.3 (1.1)

* Psychotropic medication: antidepressants, mood regulators, hypnotics, antipsychotics

^#^ First MBI measurement

11% of the participants were MBI-free at baseline (*n* = 23), with most having 2 or 3 MBI domains present (*n* = 105, 52%). The prevalence of MBI domains was as follows (in descending order): affective dysregulation, *n* = 149, 74.9%; decreased motivation, *n* = 107, 53.8%; impulse dyscontrol, *n* = 101, 50.8%; social inappropriateness, *n* = 42, 21.1%; abnormal perception, *n* = 29, 14.6%.

Gait speed was stable during the 24-month follow-up with a non-significant overall change of 0.004 m/s (95% CI −0.055; 0.047), adjusted by covariates.

**Fig. 1:**
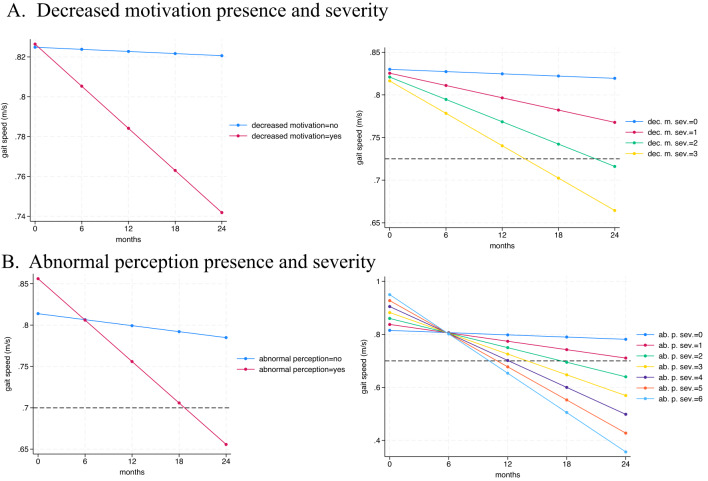
Gait speed decline according to MBI domains of decreased motivation presence and severity at baseline.Panel (A) MBI domain of decreased motivation. Panel (B) MBI domain of abnormal perception. Legend: dec. m. sev.= decreased motivation severity score. ab. p. sev.= abnormal perception severity score. Black dashed line = minimal clinically important difference

legend: dec. m. sev.= decreased motivation severity score. ab. p. sev.= abnormal perception severity score. Black dashed line = minimal clinically important difference

Participants with the MBI domain of decreased motivation at baseline exhibited a steeper decline in gait speed over the follow-up period than those without decreased motivation (*p* = 0.037). For each point of higher severity in the decreased motivation domain at baseline, the participant’s gait speed declined by 0.047 m/s more than those without decreased motivation (95% CI: −0.092 to −0.002) (*p* = 0.043) over 24 months of follow-up (Table 2; Fig. 1). The p values for the MBI x time x age interactions were all non-significant. Supplementary material 1.

**Table 2:** Mixed models with gait speed as the outcome

n=223	Coefficient	p-value	95% CI
**Baseline**			
MBI domains presence			
*Decreased motivation*	*−0.0033*	*0.037*	*−0.0065 −0.0002*
Emotional/affective dysregulation	0.0027	0.112	−0.0006 0.0061
Impulse dyscontrol	−0.0014	0.396	−0.0045 0.0018
Social inappropriateness	0.0001	0.966	−0.0036 0.0038
Abnormal perception	−0.004	0.122	−0.0090 0.0011
MBI domains severity			
*Decreased motivation*	*−0.002*	*0.043*	*−0.0039 −0.0001*
Emotional/affective dysregulation	0.0003	0.513	−0.0007 0.0014
Impulse dyscontrol	−0.0005	0.322	−0.0014 0.0005
Social inappropriateness	0	0.973	−0.0023 0.0022
Abnormal perception	−0.0012	0.39	−0.0039 0.0015
**Time Dependent**			
MBI domains presence			
Decreased motivation	−0.0029	0.115	−0.0065 0.0007
Emotional/affective dysregulation	−0.0012	0.543	−0.0026 0.0050
Impulse dyscontrol	−0.0034	0.079	−0.0071 0.0004
Social inappropriateness	−0.0023	0.28	−0.0064 0.0018
*Abnormal perception*	*−0.0071*	*0.004*	*−0.0120 −0.0023*
MBI domains severity			
Decreased motivation	−0.0002	0.848	−0.0020 0.0017
Emotional/affective dysregulation	−0.0003	0.58	−0.0013 0.0007
Impulse dyscontrol	−0.0008	0.15	−0.0018 0.0003
Social inappropriateness	−0.0009	0.497	−0.0034 0.0016
*Abnormal perception*	*−0.0039*	*0.003*	*−0.0064 −0.0013*

Coefficient: adjusted between-group differences in the interaction of the exposure (MBI variable) with time, which indicates the monthly rate gait speed change in m/s of the MBI-impaired groups compared with those non-impaired.

All models adjusted for time variable (months), age, sex, education, MMSE, weight, history of major depressive disorder, number of comorbidities (from the following: hypertension, diabetes, heart failure, hypercholesterolemia, chronic kidney disease, chronic obstructive pulmonary disorder, arthritis, cancer), prescription of psychotropics (i.e., antidepressants, antipsychotics, mood-regulators, hypnotics).

Participants with abnormal perception at any time during follow-up exhibited a steeper decline in gait speed compared to their counterparts (*p* = 0.004). For each point of higher severity for abnormal perception at any time during follow-up, the participant’s gait speed declined by 0.093 m/s more than those without abnormal perception (95% CI, −0.155 to −0.032) (*p* = 0.003) over 24 months of follow-up (Table 2; Fig. 1).

**Fig. 2:**
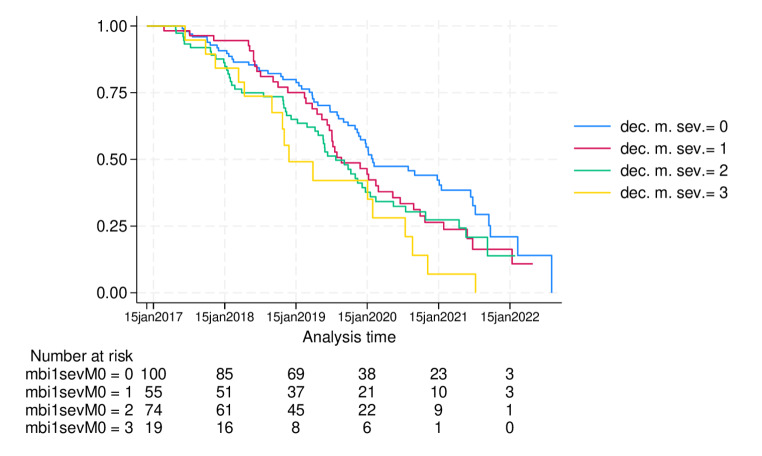
Kaplan Meier curves for frailty incidence according to the severity of decreased motivation at baseline.Legend: dec. m. sev.= decreased motivation severity score.

Among pre-frail participants at baseline, each point of higher severity for decreased motivation at baseline resulted in a 33% higher risk of incident frailty than their counterparts over a mean follow-up of 284 days (SD 346), HR = 1.33, 95% CI 1.07–1.66; *p* = 0.009. Similarly, for each point of higher severity in decreased motivation at any time during the follow-up, they showed a 39% higher risk of incident frailty than their counterparts, with an HR of 1.39 (95% CI 1.12–1.72; *p* = 0.003). (Table 3.; Fig. 2).

**Table 3:** Hazard Ratio based on Cox Models for Incident frailty

n=208, n of events= 104	HR	p-value	95% CI
**Baseline**			
MBI domains presence			
Decreased motivation	1.38	0.112	0.92 2.04
Emotional/affective dysregulation	1.14	0.573	0.71 1.81
Impulse dyscontrol	0.89	0.571	0.60 1.32
Social inappropriateness	1.03	0.887	0.64 1.65
Abnormal perception or thought content	0.77	0.375	0.42 1.37
MBI domains severity			
*Decreased motivation*	*1.33*	*0.009*	*1.07 1.65*
Emotional/affective dysregulation	1.03	0.609	0.92 1.14
Impulse dyscontrol	0.97	0.615	0.86 1.08
Social inappropriateness	0.97	0.823	0.73 1.28
Abnormal perception or thought content	0.85	0.354	0.61 1.19
**Time Dependent**			
MBI domains presence			
Decreased motivation	1.47	0.059	0.98 2.19
Emotional/affective dysregulation	1.18	0.49	0.74 1.86
Impulse dyscontrol	0.93	0.727	0.63 1.37
Social inappropriateness	0.82	0.42	0.49 1.33
Abnormal perception	0.86	0.586	0.50 1.47
MBI domains severity			
*Decreased motivation*	*1.39*	*0.003*	*1.12 1.72*
Emotional/affective dysregulation	1.08	0.179	0.96 1.19
Impulse dyscontrol	1.01	0.837	0.90 1.13
Social inappropriateness	0.93	0.634	0.70 1.24
Abnormal perception	0.92	0.632	0.67 1.27

HR hazard ratio. All models adjusted for age, sex, education, MMSE, weight, history of major depressive disorder, number of comorbidities (from the following: hypertension, diabetes, heart failure, hypercholesterolaemia, chronic kidney disease, chronic obstructive pulmonary disorder, arthritis, cancer), prescription of psychotropics (i.e., antidepressants, antipsychotics, mood-regulators, hypnotics).

## Discussion

We found a longitudinal association between the MBI domains of decreased motivation and abnormal perception and frailty. The presence and severity of the decreased motivation domain at baseline were significant predictors of a faster decline in gait speed over the follow-up period. Consistently, decreased motivation severity at baseline or as a time-dependent variable was associated with a higher risk of incident frailty phenotype. The presence and severity of the abnormal perception domain, as a time-dependent variable, were also associated with a significantly steeper decline in gait speed compared to those who were not impaired in that domain. These associations were statistically significant after adjusting for confounders. Age did not significantly modify the association between MBI and frailty in our study. The high prevalence of MBI is in line with an advanced age and frail population [23].

Previously published studies had cross-sectionally explored the association between MBI and frailty. Fan and co-authors [11] found a significant association between the MBI domains of decreased motivation, affective dysregulation, and social inappropriateness, and Fried’s frailty phenotype [3]. On the other hand, Guan and co-authors [12] found an association between the frailty index [24] and the MBI domains of decreased motivation, abnormal perception, and affective dysregulation. Guan and Chen [13] reported an association between MBI total score and dual-task gait speed. Those cross-sectional studies included younger and more robust patients than ours (mean age of 69.7 years, SD 7.6, with 35.2% robustness for the first case and 72.2 years, SD 6.5, 69.3% robustness for the second, and 72.4 years, SD 6.4 for the third). Our results align with previous studies on the link between MBI domains of decreased motivation, abnormal perception, and frailty. Furthermore, our study points towards a genesis of MBI upstream of the natural history of frailty, including among the oldest old.

Decreased motivation, a feature of apathy, has been defined as a quantitative reduction of goal-directed activity in behavioral, cognitive, emotional, or social dimensions compared to the patient’s previous level of functioning in these areas. Apathy has been considered a marker of frailty [25], with apathetic participants classified twice as likely as pre-frail and thrice as likely as frail compared to non-apathetic [26]. Apathy is characterized by cognitive, motor, and sensory impairment in initiating behavior toward a given goal and has been linked to diminished physical activity [27, 28].

At the brain level, apathy has been associated with connectivity dysfunctions between the prefrontal cortex (PFC) and the basal ganglia, which impact decision-making and motor response [25, 26, 29, 30]. For instance going through the (1) emotional and affective, (2) cognitive and (3) self-activation mechanisms [29]. In this vein, the nucleus accumbens, often disrupted in apathetic patients, is crucial for integrating emotional and behavioral information and translating emotions into motor actions [26, 31].

In individuals without major neurocognitive disorders, apathy has been associated with a diffuse loss of gray matter volume and white matter lesions in the frontal and temporal lobes [32]. Such abnormalities disrupt the planning and initiation of motor actions and are associated with reduced decreased walking speed, thus contributing to the development of frailty [26, 33–36]. Also, studies have shown vascular lesions on cerebral MRI scans of patients with multiple chronic conditions [25, 33, 37, 38], leading to slowness of gait and, therefore, increasing patients’ frailty and disability [32, 33].

In the literature, studies have shown that increased interleukin 6, C-reactive protein, and white blood cell levels were associated with frailty, apathy, disability, and walking problems [33, 39–43]. Those findings suggest that the level of inflammation could be one of the factors explaining the link between MBI and frailty. To this end, more research is needed with a mechanistic approach to assess the link between markers of inflammation and MBI.

A relationship has been established between symptoms of psychosis, such as hallucinations and delusions, and frailty severity [44]. Furthermore, lesions in the frontal lobe or the basal ganglia can be responsible for both hallucinations and apathy [45, 46].

However, the fact that not all the MBI domains were associated with frailty in our study suggests that the pathophysiological mechanisms behind each of the MBI domains may be heterogeneous. While apathy and abnormal perception may share common pathways with frailty, social inappropriateness, affective dysregulation, and impulse dyscontrol may indirectly contribute to frailty through social isolation, reduced social support, poor adherence to healthy habits, and risky behaviors.

Our study’s strengths include being the first to approach this question with a longitudinal design, and with a representation of very old, non-demented individuals from a real-life frailty clinic. Consistency with previous cross-sectional studies that involved younger patients, supports the robustness of the observed relationship between MBI and frailty, across different age profiles, and using two frailty metrics. A limitation of our study is that we did not use the MBI-C scale to assess the MBI, as the COGFRAIL study occurred before the publication of this scale. However, we have utilized an accepted algorithm that enables a close translation between the NPI and MBI-C scales [47], which are not equivalent but correlated [8, 48]. To get closer to the ISTAART MBI criteria, we adjusted our models for patients with a self-report of depression, but we could not verify that the NPS were later-life emergent and persistent for at least 6 months (NPI‐Q spans 1 month before the interview). Nonetheless, even if our results could be inaccurate due to classification error, we maximized the possibility of capturing symptomatology at any of the times points with a six-month interval by using time-varying models. Another limitation is that we could not clearly identify participants with major psychiatric disorder, but we used the self-report of depression as the best available proxy [49, 50]. Finally, when interpreting our findings the reader should bear in mind that our population of very old pre-frail and frail adults with MCI, lacked robust and cognitively normal subjects [14]. Older adults with MCI tend to over-report their physical activity levels (with a mean difference of 13%), which could potentially have a slight impact on the frailty classification status [51–53]. The fact of testing multiple hypotheses simultaneously could raise concerns of type I error; however, several factors support the validity of our results: (1) decreased motivation was consistently linked with both frailty measures, (2) abnormal perception showed the largest effect sizes for gait speed, (3) comparisons were planned as part of the study design; all of which reassures against random findings.

In the future, we envision verifying our findings among a population that includes younger subjects using the MBI-C scale, along with the incorporation of imaging and Geroscience biomarkers, which will allow us to delve further into the association between frailty and MBI.

## Conclusion

In conclusion, the study found that MBI decreased motivation and abnormal perception domains are associated with incident frailty in a very aged population with mild cognitive impairment. These results provide valuable insights into the dynamic relationship between neuropsychiatric changes and physical decline in very old adults [54]. Comprehensive care can benefit aging populations most significantly by targeting the neurobehavioral domain, and holistic interventions for MBI are urgently needed.

## Supplementary Information

Below is the link to the electronic supplementary material.

Supplementary Material 1

## Data Availability

Data requests are accepted at ihuos\_inspiredataaccess@chu-toulouse.fr. The STATA code used for this study is available upon request from the corresponding author.
