# Supplementary material for: Mild behavioral impairment is longitudinally associated with frailty in very old adults with MCI: insights from COGFRAIL
Source: Aging Clin Exp Res. 2026 Feb 3;38(1):64. doi: 10.1007/s40520-025-03309-9 (PMC12872730; doi:10.1007/s40520-025-03309-9)
Supplement: Supplementary file 1 — Supplementary Material 1 [file 40520_2025_3309_MOESM1_ESM.docx]

**Supplementary material 1**

Coefficients for the MBI x time x age interaction in the linear mixed-effect models for gait speed with time-varying MBI variables.

|  | Domain | coeff. | p | 95%CI |  |
| --- | --- | --- | --- | --- | --- |
| Domain 1 | Apathy | 0.00001 | 0.962 | -0.00042 | 0.00044 |
| Domain 2 | Affective dysregulation | 0.00002 | 0.840 | -0.00017 | 0.00021 |
| Domain 3 | Impulsive dyscontrol | -0.00009 | 0.413 | -0.00030 | 0.00012 |
| Domain 4 | Social Inappropriateness | -0.00027 | 0.283 | -0.00077 | 0.00023 |
| Domain 5 | Abnormal perception | 0.00008 | 0.709 | -0.00034 | 0.00050 |
